# A Machine Learning Approach Reveals a Microbiota Signature for Infection with Mycobacterium avium subsp. *paratuberculosis* in Cattle

**DOI:** 10.1128/spectrum.03134-22

**Published:** 2023-01-19

**Authors:** Sang-Mok Lee, Hong-Tae Park, Seojoung Park, Jun Ho Lee, Danil Kim, Han Sang Yoo, Donghyuk Kim

**Affiliations:** a School of Energy and Chemical Engineering, Ulsan National Institute of Science and Technology, Ulsan, Republic of Korea; b Department of Infectious Diseases, College of Veterinary Medicine, Seoul National University, Seoul, Republic of Korea; c Department of Farm Animal Medicine, College of Veterinary Medicine, Seoul National University, Seoul, Republic of Korea; University of Prince Edward Island

**Keywords:** *Mycobacterium avium* subsp. *paratuberculosis*, gut microbiota signature, machine learning-based predictive model, 16S rRNA sequencing, feature selection

## Abstract

Although Mycobacterium avium subsp. *paratuberculosis* (MAP) has threatened public health and the livestock industry, the current diagnostic tools (e.g., fecal PCR and enzyme-linked immunosorbent assay [ELISA]) for MAP infection have some limitations, such as inconsistent results due to intermittent bacterial shedding or low sensitivity during the early stage of infection. Therefore, this study aimed to develop a novel biomarker focusing on elucidating the gut microbial signature of MAP-positive ruminants, since the clinical signs of MAP infection are closely related to dysbiosis. 16S rRNA-based gut microbial community analysis revealed both a decrease in microbial diversity and the emergence of several distinct taxa following MAP infection. To determine the discriminant taxa diagnostic of MAP infection, machine learning-based feature selection and predictive model construction were applied to taxon abundance data or their transformed derivatives. The selected taxa, such as Clostridioides (formerly Clostridium) difficile, were used to build models using a support vector machine, linear support vector classification, *k*-nearest neighbor, and random forest with 10-fold cross-validation. The receiver operating characteristic-area under the curve (ROC-AUC) analysis of the models revealed their high accuracy, up to approximately 96%. Collectively, taxonomic signatures of cattle gut microbiotas according to MAP infection status could be identified by feature selection tools and applied to establish a predictive model for the infection state.

**IMPORTANCE** Due to the limitations, such as intermittent bacterial shedding or poor sensitivity, of the current diagnostic tools for Johne’s disease, novel biomarkers are urgently needed to aid control of the disease. Here, we explored the fecal microbiota of Johne’s disease-affected cattle and tried to discover distinct microbial characteristics which have the potential to be novel noninvasive biomarkers. Through 16S rRNA sequencing and machine learning approaches, a dozen taxa were selected as taxonomic signatures to discriminate the disease state. In addition, when constructing predictive models using relative abundance data of the corresponding taxa, the models showed high accuracy for classification, even including animals with subclinical infection. Thus, our study suggested novel noninvasive microbiological biomarkers that are robustly expressed regardless of subclinical infection and the applicability of machine learning for diagnosis of Johne’s disease.

## INTRODUCTION

Mycobacterium avium subspecies *paratuberculosis* (MAP) is an infectious pathogen causing Johne’s disease (JD) or paratuberculosis (PTB) in ruminants (1). Animals infected with MAP suffer from chronic enteritis and diarrhea, which causes decreases in productivity, such as milk yield loss and infertility (1), and even leads to death (2). Although MAP infection can cause devastating effects, its long incubation period makes it difficult to eradicate (3). Clinically symptomatic animals are the “tip of the iceberg,” which indicates that many other individuals are in a silent or subclinical state, having acquired infection by herd transmission events (4). Additionally, MAP has been identified as a zoonotic pathogen (5) that plays a pivotal role in the pathogenesis of various diseases (6, 7). Therefore, diagnostic tools to detect MAP infection are crucial to prevent damage to the livestock industry and public health.

To overcome the limitation of high costs and time-consuming diagnoses based on bacterial culture, several culture-independent methods have been developed, such as fecal PCR (8) and enzyme-linked immunosorbent assay (ELISA) (9). Fecal PCR can detect the infectious state with high sensitivity, but there are still challenges, such as PCR inhibitors in feces (10), insufficient primer specificity (11), and low-level intermittent shedding during the subclinical stage (12). ELISA kits for MAP detection using serum or milk samples are available, but their low sensitivity during the early stage of infection remains a limitation. Hence, the need for alternative diagnostic tools with novel approaches for detecting MAP infection is urgent.

The gut of a eukaryotic host harbors a complex and dynamic population of various microorganisms, referred to as the microbiota. As the composition of the gut microbiota is modulated by various factors, such as diet, antibiotics, and disease state (13), many researchers have suggested novel biomarkers to indicate specific conditions by forming indices with populations of differentially abundant taxa (e.g., the *Firmicutes*/*Bacteroidetes* [F/B] ratio [14, 15]) or identifying closely associated bacteria (e.g., Faecalibacterium prausnitzii [16]). As the main route of MAP infection is fecal-oral transmission (1) and its primary clinical sign is granulomatous diarrhea, which is closely related to dysbiosis, investigating the distinct features of the gut microbiota of MAP-infected individuals may provide insight into biomarker discovery. However, the structure of the microbial community and the microbial signatures of JD are still poorly understood.

Machine learning (ML) has been widely applied in biological studies since the quantity of data generation began to rapidly increase with advances in next-generation sequencing technologies (17). After the explosion of biological data, data mining to find patterns and extract useful biological insights from multiple types of data sets has become a bottleneck (18). Various ML algorithms have demonstrated their usefulness in integrating heterogeneous biological data with a noisy nature (19). For example, ML-based diagnostic models for general dysbiosis states (20) or diseases such as Crohn’s disease (CD) (21) have succeeded in classifying abnormal states with high accuracy. Data regarding bacterial abundance in microbiotas are also used as input for ML algorithms to train the predictive model (22).

This study aimed to capture the microbial signature of MAP infection, thereby developing a classification model to aid diagnosis and discover novel MAP-associated biomarkers using microbial composition data. First, the microbial diversity and taxonomic profile in the gut microbiota of MAP-infected cattle were explored based on 16S rRNA sequencing. Subsequently, five feature selection tools, including ridge regression, LASSO, ElasticNet, Feature Selector, and the filter method, were used to determine closely correlated features using high-dimensional microbial abundance data. Furthermore, ML-based predictive models for MAP infection using linear support vector classification (LinearSVC), k-nearest neighbor (KNN), random forest, and support vector machine (SVM) were constructed, and their performances were compared. Taken together, the results predicted that taxonomic signatures of MAP-positive cattle could be identified by feature selection tools, and the ML classification model could predict the infection state with high accuracy using these signatures.

## RESULTS

### MAP infection modulates the gut microbiota of cattle in the direction of decreasing microbial diversity.

**FIG 1:**
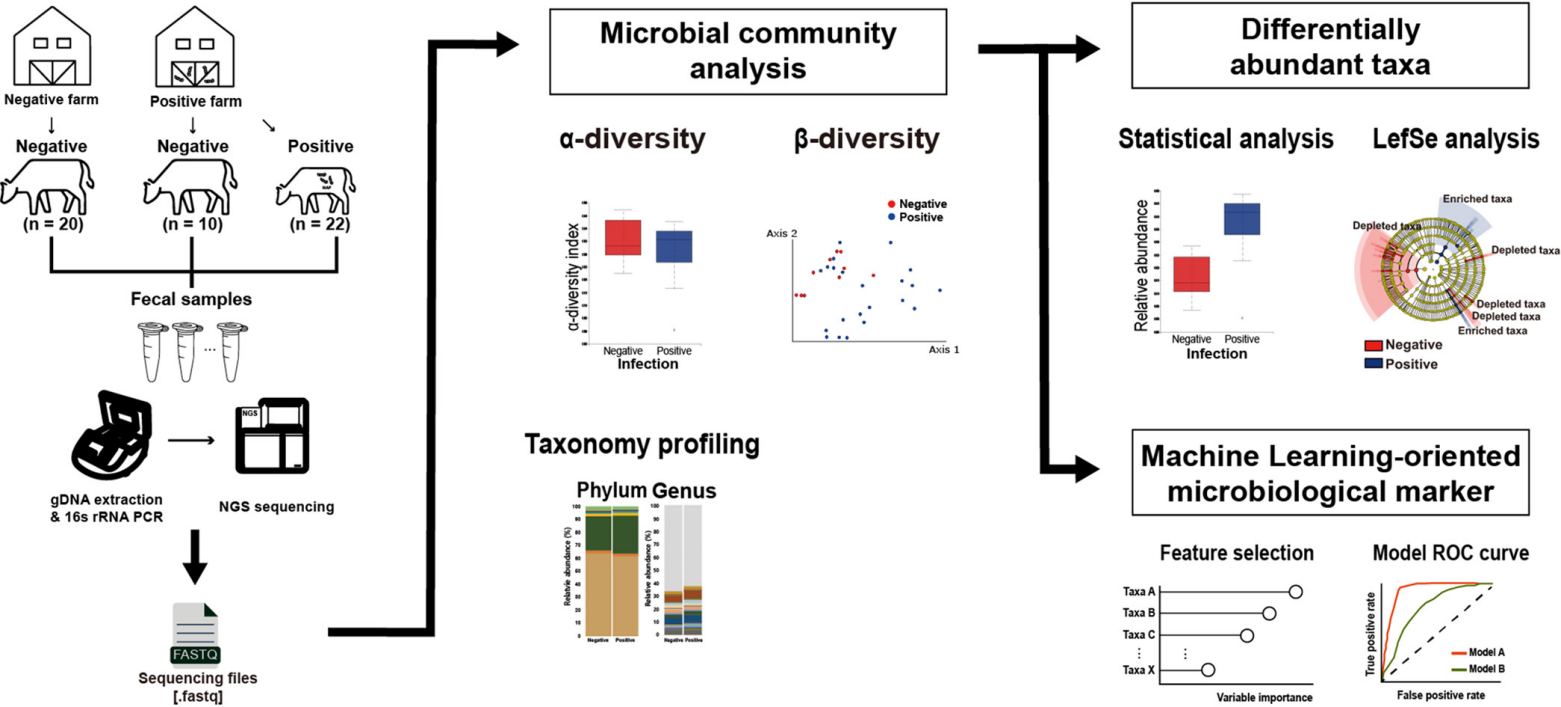
Overview of the analysis. The experiment was conducted using fecal samples from 52 cattle (negative, 30; positive, 22) divided into two groups by MAP infection. Microbial diversities (alpha and beta) and taxonomy profiles of the microbiotas in all samples were explored and compared by group. To determine the microbial features associated with MAP infection, significantly different taxa were identified by statistical analysis and LEfSe analysis based on the relative abundances of each taxon. Several taxa that contributed to classifying MAP infection were selected using dimensionality reduction tools, and their discriminating potential for the MAP infection classifier was validated using ROC curve analysis.

To determine the impact of MAP infection on the gut microbial community and to determine distinct microbiological features reflecting infection, microbial community analysis using the fecal microbiotas of 22 MAP-positive and 30 MAP-negative cattle was conducted via 16S rRNA sequencing (Fig. 1). On average, 19,469 ± 6,130 paired-end reads were obtained for each sample from a total of 1,012,401 reads.

**FIG 2:**
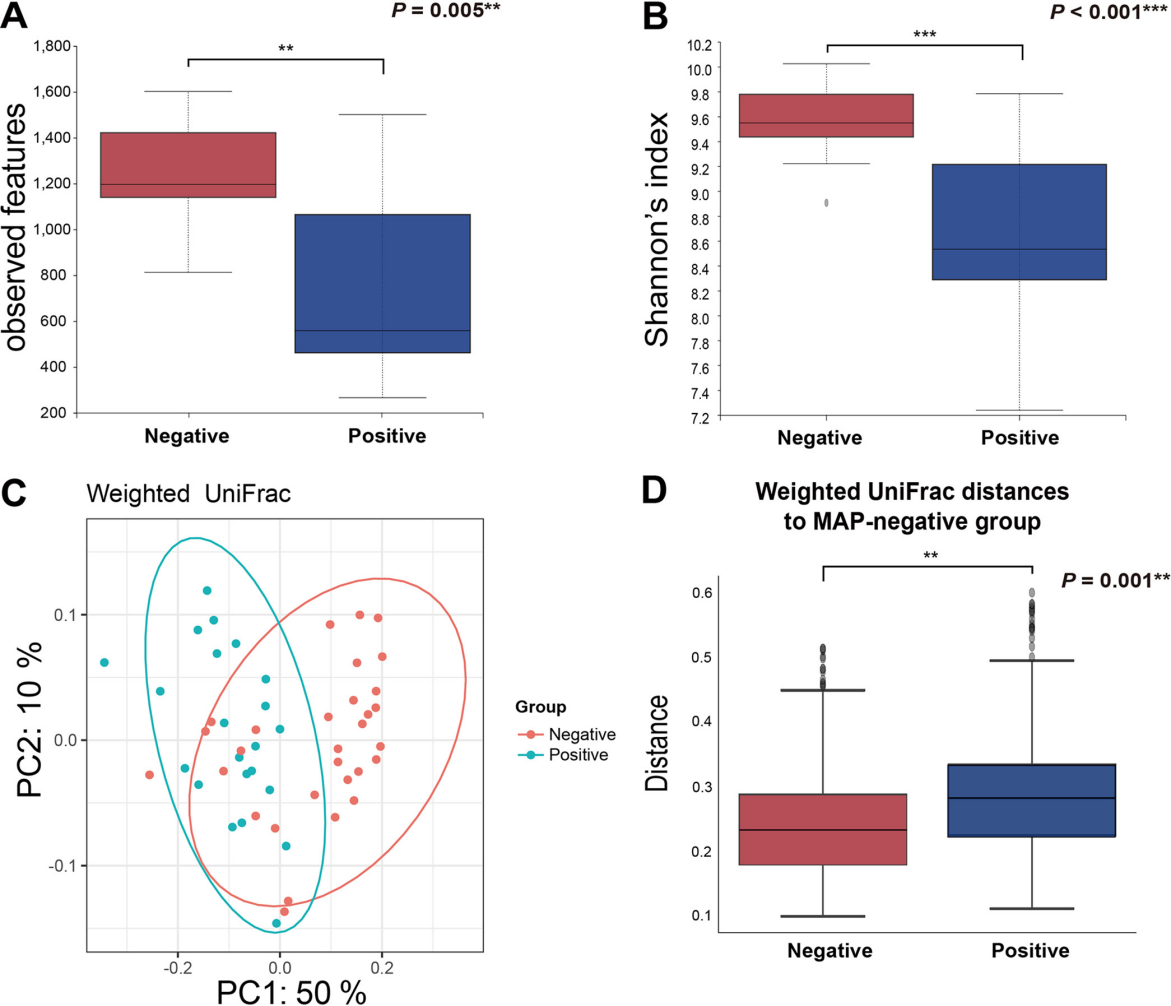
Changes in microbial diversity indices of fecal microbiotas by MAP infection. (A and B) Alpha diversity indices for richness (A) (observed features) and diversity (B) (Shannon’s index). (C and D) Beta diversity indices based on weighted UniFrac distances visualized in the form of PCoA plots that demonstrate significant differences by their distances from each sample between groups.

First, differences in various alpha and beta diversity indices related to MAP infection were investigated. The microbial richness (observed features; *P = *0.005) (Fig. 2A) and diversity (Shannon’s index; *P < *0.001) (Fig. 2B) were significantly decreased by MAP infection. Likewise, other alpha diversity indices for microbial evenness (Pielou’s evenness; *P < *0.001) (see Fig. S2A in the supplemental material) and diversity (Faith’s phylogenetic diversity [PD] [*P < *0.001] and Simpson’s index [*P < *0.001]) (Fig. S2B and C) showed decreased patterns. Subsequently, principal coordinate analysis (PCoA) performed based on weighted UniFrac distances (Fig. 2C) and the distribution of the distances between samples in the MAP-negative and -positive groups (Fig. 2D) (permutational multivariate analysis of variance (PERMANOVA), 999 permutations; *P = *0.001) revealed that the gut microbial communities were significantly altered in response to MAP infection. PCoA plots and distance distribution (PERMANOVA, 999 permutations; *P = *0.001) based on unweighted UniFrac distances also showed apparent clustering of the microbiotas (Fig. S2). The analysis suggested that MAP infection diminished the microbial diversity of the gut microbiota in cattle.

### Several microbial taxa showed distinct population changes in response to MAP infection.

**FIG 3:**
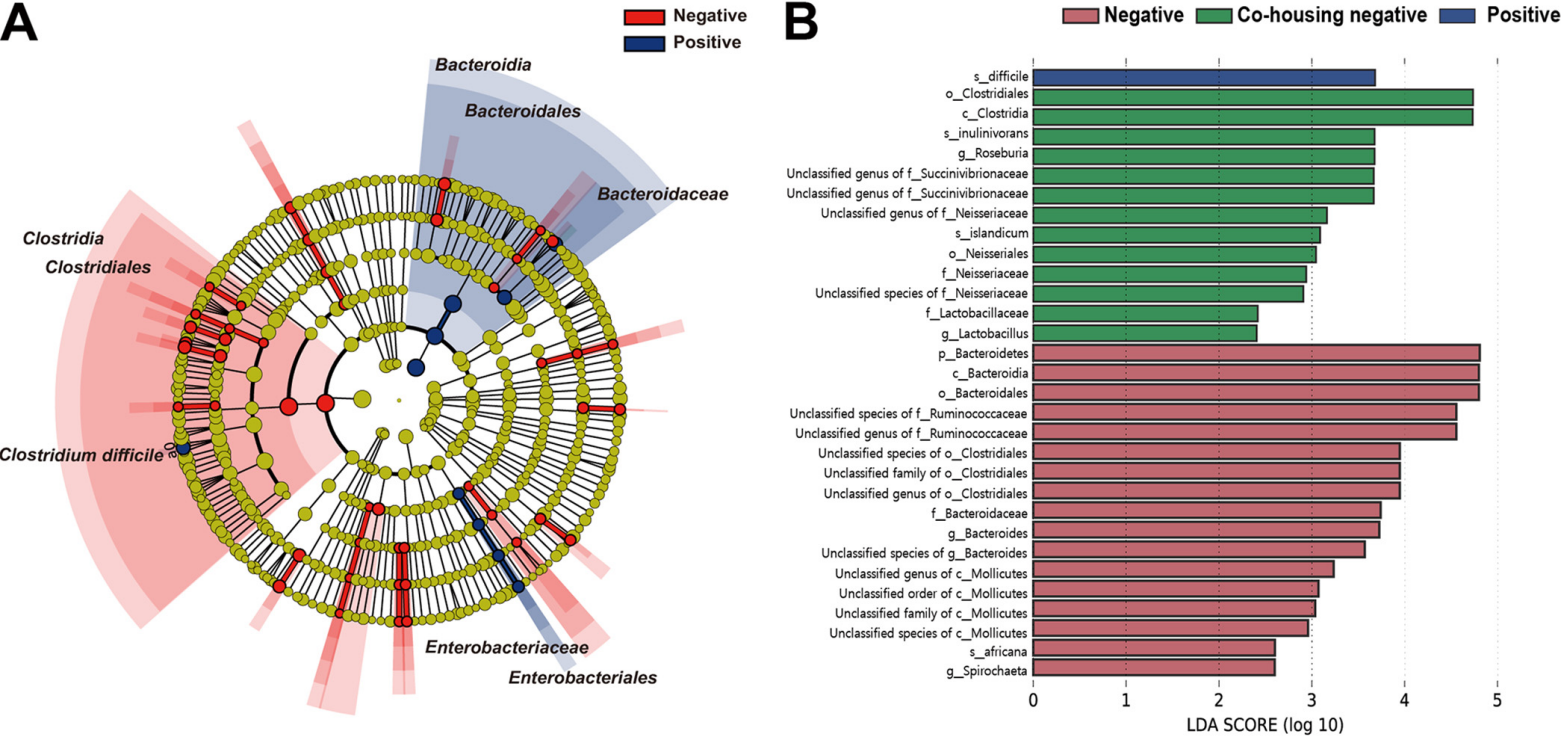
LEfSe analysis for determining differentially abundant taxa by MAP infection. (A) Cladogram generated by LEfSe demonstrating differential abundances of taxa (LDA > 3.0); (B) bar graph showing LDA scores for the negative-farm group, the negative group cohoused on the positive farm, and the positive group (positive farm). Only taxa meeting the LDA significance threshold are shown in the bar chart (LDA > 2.0).

The relative abundance values of microbial taxa in each group were investigated. To identify taxa specific for MAP infection, linear discriminant analysis (LDA) effect size (LEfSe) analysis was conducted by the MAP-negative/-positive group (Fig. 3A). Several differentially abundant taxa were identified at various taxonomic levels. The circular cladogram for LEfSe (threshold, LDA score > 3.0) indicated that the classes *Clostridia* and *Bacteroidia* were the discriminant taxa of the MAP-negative and MAP-positive groups, respectively. Additionally, the amplicon sequence variants (ASVs) assigned to the class *Clostridia* and the order *Clostridiales* were significantly enriched in the MAP-negative groups, whereas the MAP-positive group was characterized by a significantly high abundance of ASVs assigned to the orders *Bacteroidales* and *Enterobacteriales*, the families *Bacteroidaceae* and *Enterobacteriaceae*, and the species Clostridioides (formerly Clostridium) difficile. The effects of the differential composition of the taxonomic profile caused by MAP infection on the metabolic function of the gut metagenome were also investigated. The abundances of Kyoto Encyclopedia of Genes and Genome (KEGG) pathways predicted by PICRUSt2 software were used to perform LEfSe analysis to determine differentially abundant pathways for each group (Fig. S3A). Among the 25,961 ASVs, 7 were above the maximum NSTI (nearest-sequenced taxon index) cutoff of 2.0 and were therefore removed from the downstream analysis. Moreover, the bovine gut microbiome was also predicted using CowPI (23), a functional inference tool specific to rumen microbiomes (Fig. S3B). Notably, pathways such as “metabolism” and “amino acid metabolism” were commonly predicted at significantly higher levels in the MAP-negative group, whereas “environmental information processing,” “membrane transport,” and “transporters” were commonly predicted at significantly higher levels in the MAP-positive group (threshold, LDA score > 3.0).

### Feature selection identified microbial taxa closely correlated with MAP infection.

To investigate major informative taxa that were closely correlated with MAP infection and to develop a predictive model, five feature selection methods (ridge regression, LASSO, ElasticNet, Feature Selector, and the filter method) were applied to the data set of relative abundance values for all taxa (referred to as the raw set). Two additional data sets, each of which consists of original values to the power of 1.5 (1.5 power set) and constant *e* to the original values (Exp set), were prepared to increase the variances of the original data set and were used together for further analysis.

**FIG 4:**
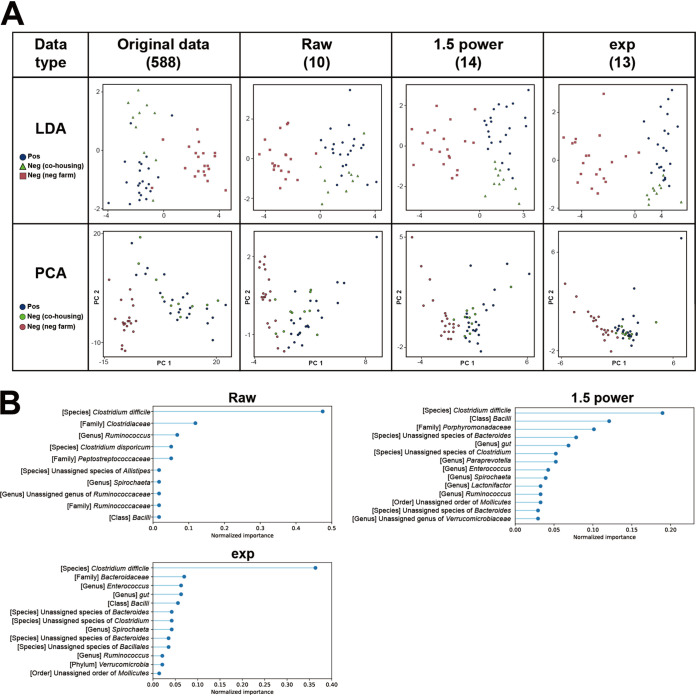
Feature selection of microbial abundance data sets by Feature Selector with three different types of transformed values. (A) LDA and PCA plots for the original relative abundance data set and different transformation types (raw, 1.5 power, and Exp). Blue, MAP positive; green, MAP negative cohoused on a positive farm; red, MAP negative on a negative farm. The values in parentheses indicate the number of selected features. (B) Variable-importance plots for the selected microbial features of each transformation type.

First, several taxa with constant and quasi-constant values among 52 samples were removed from each data set. The threshold for the quasi-constant was set to 0.00005 based on the variance among the samples. By that, from 588 taxa, the number of remaining taxa for the raw set was 478, while those for the 1.5 power and Exp sets were 387 and 480, respectively (Fig. S4). Subsequently, using the five feature selection methods, feature selection of each data set was conducted from the remaining taxa to classify individuals into three groups: MAP positive, MAP negative cohoused on the positive farm, and MAP negative on the negative farm. LDA and principal-component analysis (PCA) plots were generated from the selected features of each case to visualize its discriminating pattern. The clustering performances among them were quantitatively compared using the Calinski-Harabasz index and the silhouette score (Table S2). In the cases of the raw and 1.5 power sets, ElasticNet showed the highest scores for both indicators using 143 and 124 features, while LASSO showed the highest scores in the Exp set with 124 features. Meanwhile, although Feature Selector mostly showed the lowest scores for all cases except the Calinski-Harabasz index of the raw set, it showed apparent clusters by groups with only 12, 13, and 13 features in the raw, 1.5 power, and Exp sets, respectively (Fig. 4A). That is, only approximately a dozen features were required to explain 98% of the whole data set, since the parameter threshold for the cumulative importance of Feature Selector was 0.98. Indeed, distributions of dots in the PCA plots based on Feature Selector-originated features were similar to those of the original data set. The selected important features for each data type were plotted (Fig. 4B). Surprisingly, the most important feature for all data types was the relative abundance data of C. difficile, with normalized importance values of 0.475, 0.190, and 0.364 for the raw, 1.5 power, and Exp sets, respectively. The values for *Bacilli* (raw, 0.017; 1.5 power, 0.121; Exp, 0.056) and *Ruminococcus* (raw, 0.068; 1.5 power, 0.033; Exp, 0.021) were commonly observed from all data types as well. In addition, for the raw set, *Clostridiaceae* (0.119), *Peptostreptococcaceae* (0.051), and *Ruminococcaceae* (0.017) at the family level, *Spirochaeta* (0.017) and an unassigned genus of *Ruminococcaceae* (0.017) at the genus level, and Clostridium disporicum (0.051) and an unassigned species of *Alistipes* (0.017) at the species level were additionally selected. For the 1.5 power set, these features were 1 order (unassigned order of *Mollicutes*, 0.033), 1 family (*Porphyromonadaceae*, 0.101), 6 genera (*Lactonifactor*, 0.033; unassigned genus of *Verrucomicrobiaceae*, 0.029; *Paraprevotella*, 0.052; gut, 0.069; *Enterococcus*, 0.042; *Spirochaeta*, 0.039), and 3 species (unassigned species of *Clostridium*, 0.052; two unassigned species of *Bacteroides*, 0.029 and 0.078). For Exp, these features were 1 phylum (*Verrucomicrobia*, 0.021), 1 order (unassigned order of *Mollicutes*, 0.014), 1 family (*Bacteroidaceae*, 0.070), 3 genera (gut, 0.063; *Enterococcus*, 0.063; *Spirochaeta*, 0.042), and 4 species (unassigned species of *Bacillales*, 0.035; unassigned species of *Clostridium*, 0.042; two unassigned species of *Bacteroides*, 0.035 and 0.042). Considering the number of selected features and the clustering performances among tools, Feature Selector was chosen to build a predictive model for MAP infection.

### The classification model based on the selected features has the potential to be a good predictor of MAP infection.

**FIG 5:**
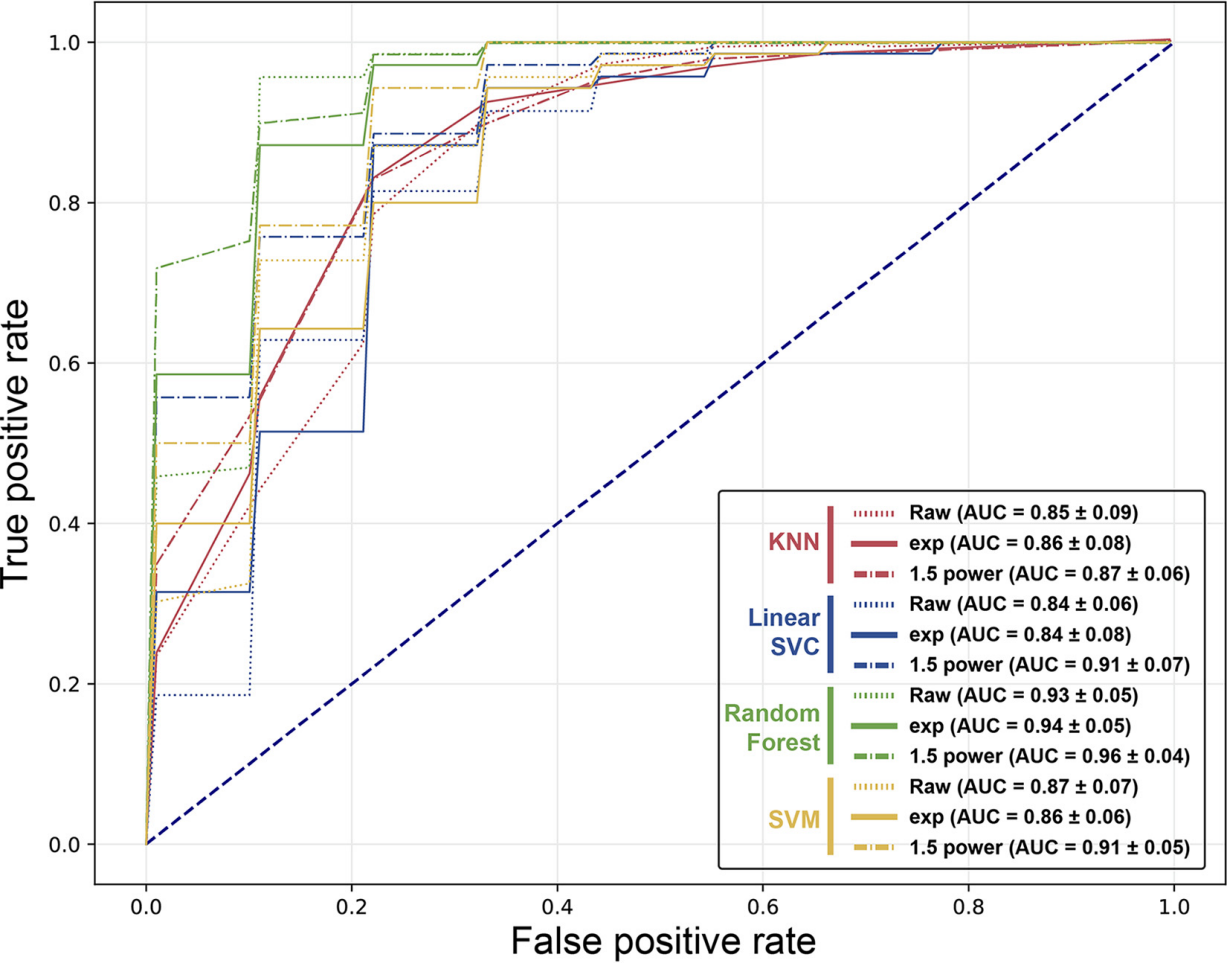
ROC curves and AUC for MAP infection-predictive models. The models were constructed using four different algorithms (KNN, LinearSVC, random forest, and SVM) with three types of data. The numbers in parentheses indicate the means and standard deviations (SD) of the AUC for each case. All ROC curves and their AUC values were averaged over 10 repetitions of 10-fold cross-validation.

Using the set of selected features by Feature Selector, machine learning models for classification of the infection state were designed and their performances were investigated. The models were built by applying four different algorithms: KNN (*k* = *3*), LinearSVC, random forest classifier, and SVM. The data were split in a ratio of 80:20 for training and testing purposes for the algorithms with 10-fold cross-validation. To assess the performances, the precision, recall, F1 score, and receiver operating characteristic-area under the curve (ROC-AUC) among the models were monitored (Fig. 5 and Table 1). In terms of predictive performance, it was observed that all indices reached at least 0.75. Notably, the best prediction results were achieved with random forest. When focusing on the AUC values, the values of 1.5 power were higher than the other data for all algorithms. Collectively, the best performance was obtained with random forest using 1.5 power data with an AUC of 0.96 for its mean value of 10-fold cross-validation. The model accuracy and AUC values of random forest models combined with the diagnosis results of fecal PCR and serum ELISA were additionally investigated, although there was no significant difference among the cases (Table S3).

**TABLE 1:** Classification performance metrics for each MAP infection-predictive model^*a*^

Algorithm	Data set	Precision	Recall	F1 score	AUC
KNN (*k* = 3)	Raw	0.81 ± 0.11	0.79 ± 0.11	0.79 ± 0.11	0.86 ± 0.09
	1.5 power	0.80 ± 0.06	0.78 ± 0.05	0.77 ± 0.06	0.87 ± 0.06
	Exp	0.83 ± 0.11	0.82 ± 0.11	0.82 ± 0.11	0.86 ± 0.08
					
LinearSVC	Raw	0.76 ± 0.11	0.75 ± 0.10	0.75 ± 0.10	0.84 ± 0.06
	1.5 power	0.86 ± 0.09	0.84 ± 0.09	0.84 ± 0.09	0.91 ± 0.07
	Exp	0.78 ± 0.06	0.76 ± 0.07	0.75 ± 0.07	0.84 ± 0.08
					
Random forest	Raw	0.90 ± 0.07	0.89 ± 0.08	0.89 ± 0.08	0.93 ± 0.05
	1.5 power	0.88 ± 0.05	0.84 ± 0.08	0.84 ± 0.08	0.96 ± 0.04
	Exp	0.88 ± 0.07	0.86 ± 0.07	0.85 ± 0.08	0.94 ± 0.05
					
SVM	Raw	0.80 ± 0.09	0.77 ± 0.08	0.77 ± 0.08	0.87 ± 0.07
	1.5 power	0.82 ± 0.07	0.80 ± 0.08	0.79 ± 0.09	0.91 ± 0.05
	Exp	0.80 ± 0.04	0.79 ± 0.04	0.79 ± 0.05	0.86 ± 0.06

a For each experiment, the precision, recall, F1 score, and AUC value of the ROC curves were considered to quantify the performance. Values are means and SD for the predictive model that applied 10-fold cross-validation (training set, *n* = 49; testing set, *n* = 5) based on the labeled information for each sample.

## DISCUSSION

Since MAP has been suspected as a productivity-reducing and/or zoonotic agent of various diseases in both ruminants and humans (24–26), accurate and rapid detection of its infection is crucial for controlling MAP-related diseases. Although several diagnostic tools, such as bacterial culture, fecal PCR, and ELISA, have been used to identify MAP infection, the alternative methods are still needed due to their time-consuming or false-negative nature. Meanwhile, the representative symptoms of the infection, such as chronic diarrhea and impairment of nutrition absorption, occur in the intestine, and the microbial community living there may be intrinsically involved in the occurrence, symptoms, and outcome of the infection. In this study, the fecal microbiota of MAP-infected cattle was investigated, and the prediction of MAP infection was carried out with machine learning models using the relative abundance values of the assigned microbial taxa.

The fecal microbiotas of MAP-infected cattle revealed significant changes in microbial richness, diversity, microbial taxon composition, and predicted metagenomes compared to those of noninfected cattle. Considering that numerous studies have reported that the microbial diversity in microbiotas is a representative characteristic of gut health status (27–29), the decreased values of overall alpha diversity indices and distinct clusters corresponding to MAP infection imply that the pathogen may induce dysbiosis. Additionally, several taxa showed distinct modulation of their population by the pathogen. Indeed, several studies reported that some taxa have significant changes in their abundance in the gut of MAP-infected individuals. For instance, C. difficile, *Bacilli*, and *Ruminococcus* were the representative taxa identified as distinct taxa according to MAP infection by feature selection. The close association of C. difficile with MAP infection was confirmed by LEfSe, by the fact that the bacterium was differentially abundant in the MAP-infected group, and by the results selected as the most important variables by feature selection. Interestingly, it was reported that both MAP and C. difficile provoked CD, whose symptoms are similar to those of JD (30, 31). Moreover, it was reported that the genus *Clostridium* showed a positive correlation with histopathology scores in MAP-infected calves (32).

In the case of *Bacilli*, although there are few studies investigating the relationship between *Bacilli* and JD, the increased population (percent relative abundance) of its lower taxonomic levels, such as the family *Bacillaceae* (Table S1) (negative, 0.68 ± 0.93; positive, 2.80 ± 1.89; Mann-Whitney U test, *P < *0.001), was reported in the case of dextran sulfate sodium-induced colitis (33).

Meanwhile, *Ruminococcus*, belonging to the family *Lachnospiraceae*, was significantly enriched in the MAP-infected group (Table S1) (negative, 0.55 ± 0.85; positive, 2.24 ± 1.41; Mann-Whitney U test, *P < *0.001). This genus has been reported for its mucin-degrading ability, and several murolytic species belonging to the genus were enriched in the gut of CD and ulcerative colitis patients (34, 35). Therefore, it is suggested that these three taxa, which were selected by feature selection using all three different values of relative abundance and their transformants, may be the keystone taxa for MAP infection.

In addition, other taxa reported as being specific to MAP infection were also observed to have distinct modulation in this study. A previous study reported that a logistic model was built using four distinct taxa to distinguish the fecal microbiota of MAP-infected calves from the noninfected group (32). Similar to that study, *Paraprevotellaceae* were significantly enriched in MAP-infected animals (negative, 1.09 ± 0.82; positive, 1.66 ± 0.62; Mann-Whitney U test, *P < *0.01) and positively correlated with the ELISA sample/positive (S/P) ratio (Pearson’s correlation coefficient [*r*] = 0.670, *P < *0.001) in this study. Although there was no significance in statistical analyses, *Faecalibacterium* was detected in only four individuals in the MAP-infected group and was depleted in all noninfected individuals. *Akkermansia* had a tendency to decrease with MAP infection (negative, 1.00 ± 0.64; positive, 0.75 ± 0.92; Mann-Whitney U test, *P = *0.0584). In the case of *Planococcaceae*, no population was observed in any MAP-infected animals in this study. In addition, the genera *Alistipes* (negative, 4.65 ± 1.52; positive, 2.94 ± 0.86; Mann-Whitney U test, *P < *0.001) and *Paraprevotella* (negative, 0.13 ± 0.15; positive, 0.08 ± 0.11; Mann-Whitney U test, *P < *0.001) were significantly decreased by MAP infection in this study, which is consistent with other studies (36, 37), whereas the overrepresentation of *Firmicutes* (negative, 56.18 ± 7.09; positive, 62.83 ± 7.33; Mann-Whitney U test, *P < *0.01), *Enterococcus* (negative, 0.03 ± 0.06; positive, 0.07 ± 0.05; Mann-Whitney U test, *P < *0.001), and Streptococcus (negative, 0.03 ± 0.09; positive, 0.08 ± 0.12; Mann-Whitney U test, *P < *0.05) in the MAP-infected group was observed, which was also reported for MAP-infected animals and humans (37, 38). In the case of *Actinobacteria*, there is controversy regarding its population change due to MAP infection. In this study, there was a significant increase in the MAP-infected group, although the highest value for its relative abundance was under 3% (negative, 0.25 ± 0.40; positive, 0.79 ± 0.62; Mann-Whitney U test, *P < *0.001), contrary to the result of up to 30% abundance in other studies (36, 39). Other dysbiosis-associated taxa identified in CD patients or diarrheic calves, such as *Enterobacteriaceae* (negative, 0.00 ± 0.1; positive, 0.11 ± 0.21; Mann-Whitney U test, *P < *0.001) and *Fusobacteriaceae* (detected in only two individuals in the MAP-positive group), were also overrepresented or detected only in MAP-positive groups, while the underrepresentation of *Porphyromonadaceae* (negative, 0.28 ± 0.13; positive, 0.17 ± 0.18; Mann-Whitney U test, *P < *0.05) was also observed (40, 41).

Microbial composition-based metagenome prediction suggested that MAP infection might modulate the gut metagenome to upregulate pathways for sensing and responding to the extracellular environment. Furthermore, the pathway representing amino acid-related metabolism was downregulated by the pathogen. Likewise, it was reported that there was a significant alteration in the metabolism of amino acids within the MAP-infected group (32). These results suggest that the biosynthesis and degradation of various amino acids may be crucial factors in dysbiosis, such as JD. Collectively, the microbiota perturbation associated with MAP infection and its subsequent metagenomic modulation corresponded well to the aforementioned previous studies. Thus, it is likely that the microbial community of MAP-infected cattle in this study might accurately reflect the general topological state of gut microbiotas infected with MAP and that, importantly, the microbiota signature identified by feature selection is reliable as well. This signature can serve as a biomarker to compensate for the drawbacks of existing tools, since the microbial community may maintain traces of MAP infection during the latent period or intermittent shedding.

While the best scenario of the predictive model based on the microbiota signature resulted in a high AUC value of approximately 0.96, a combination of the signature and other diagnostic results, such as the cell number of MAP estimated by fecal PCR and the ELISA S/P ratio, showed a decrease in the values of model accuracy and AUC (Table S3). This might be related to the low sensitivity of traditional diagnosis methods. Indeed, 50% of individuals in the positive group (11/20) showed low ELISA S/P ratios under a threshold of 50 (Table S1). The low sensitivity of ELISA using serum or milk samples has been pointed out as a limitation during the early stage of MAP infection (42, 43). Conversely, seven individuals in the positive group were not identified by fecal PCR, while the ELISA S/P ratio was over the threshold (Table S1). This result may be caused by the variable intermittent shedding in feces at different time points, which was already reported in other studies (44, 45). Furthermore, there were cases (e.g., sample 181.2, from the same animal as samples 181 and 181.3, and sample 188.2, from the same animal as 188 but collected in a different year) that continued to be judged MAP positive because at least one diagnostic tool identified them as positive, even though the diagnostic result for an individual was negative (Table S1). These inconsistent patterns of diagnostic results may weaken the discriminative power. These findings suggest that machine learning using microbial population data as input can be a novel approach to develop novel noninvasive biomarkers, thereby compensating for the low sensitivity of current diagnostic methods due to intermittent shedding or subclinical infection. Meanwhile, it was not possible to detect the abundance of MAP by 16S rRNA sequencing, which is due to its low proportion in the gut microbiota and the inadequate efficiency of genomic DNA extraction from lipopentapeptide-coated MAP (46, 47).

We acknowledge several limitations in our study. First, we used fecal samples, so the bacterial population of ileal mucosa in which MAP proliferates could not be directly identified (48). However, the purpose of this study was to discover a novel noninvasive biomarker for MAP infection that can be practically used on farms without the need for sacrificing animals. Fortunately, the microbiome signature identified in this study showed high accuracy for predicting the MAP infection state. Second, only two sites were used to collect the samples. Since the environment, including diet, has a great impact on the structure of the gut microbiota (49), differences among farms may contribute to the generation of distinct clusters of microbiotas. Indeed, it was observed that the cattle microbiota was clearly distinguished according to the farm in this study (Fig. S5) (PERMANOVA, 999 permutations; *P* = 0.001 for both unweighted and weighted UniFrac distances). Nonetheless, the microbiota signature found by statistical analyses and feature selection was trustworthy, since negative-farm samples and samples from negative animals cohoused on the positive farm were combined and then compared with those from the MAP-infected group. Although samples were collected from animals with various ages, parities, lactation periods, and breed types (Table S1), further study using large quantities of novel samples obtained from multiple sites to minimize the effect of within-animal variability is needed to validate the robustness of the microbiota signature and its derived predictive model, which was made using a relatively small number of samples in this study. Moreover, comparing distinct microbial features of the gut microbiome of MAP-infected cattle and those of other enteric infections, such as colibacillosis or salmonellosis, will provide an opportunity to clearly investigate the taxonomic signatures for MAP-specific pathology.

Last, although machine learning has broadened the current limited understanding of the complexity of microbiotas by detecting informative patterns in the microbial community system, the overfitting issue of the model should be pointed out. The generalizability of the model cannot be verified, since to our current knowledge there are no available sequence read archives of MAP-infected animals with accurate metadata. This issue may be solved by additive 16S rRNA sequencing of novel samples. Nevertheless, several studies on the application of machine learning models for disease diagnosis have been already reported (50, 51). In particular, machine learning with bacterial taxon information regarding the gut microbiome showed its potential for detecting cardiovascular disease (52). Likewise, the distinct microbial taxa identified by machine learning without human subjectivity and the subsequent generation of predictive models in this study showed another case for a microbiome-based machine learning approach for diagnostic screening, although comparative studies with large-scale follow-up experiments are needed.

In summary, the microbiota signature of MAP-infected cattle was investigated using both statistical analyses and machine learning algorithms. The results indicate that several specific microbial taxa that distinguish the infection state have the potential to be noninvasive biomarkers for classifying MAP infection to support the current diagnostic tools. In addition, the machine learning-based investigation of major features in the microbiota can be applied to other biomarker discovery studies for prophylactic or diagnostic use.

## MATERIALS AND METHODS

### Sample collection.

All specimens were obtained from farms that were referred to our laboratory for diagnostic testing for Johne’s disease. ELISA and fecal real-time PCR tests were used to cross-validate, and the diagnostic tests were conducted a total of three times, periodically from 2019 to 2021. To determine the infection state of animals, the animals were labeled negative only when all tests were consistently negative, and those whose tests were positive at least once were considered infected. The negative farm was initially selected because no cases of MAP infection had been reported in the quarantine system of South Korea, and additional tests were conducted for every new animal that came to the farm. Based on the results, two farms, positive and negative, were selected for this study, and a total of 52 animals were selected for analysis according to their infection status. Twenty-two dairy cows that were positive on diagnostic tests along with 10 negative cows with evenly distributed ages and parities were selected on the positive farm. On the negative farm, a total of 20 negative animals, 10 Korean native cattle (Hanwoo) and 10 Holstein dairy cows, were selected using the same criteria.

Infection with MAP was diagnosed by a commercial ELISA kit (Idexx Laboratories Inc., Westbrook, ME, USA) and a fecal real-time PCR assay that was optimized in our laboratory. ELISA was performed using serum samples according to the manufacturer’s instructions. For DNA extraction for real-time PCR, 2 g of feces was mixed with 35 mL of distilled water (DW), and the upper 5 mL was used after standing for 30 min. Fecal DNA extraction was conducted using a fecal microbe DNA extraction kit (Zymo Research, Irvine, CA, USA) according to the manufacturer’s instructions. Two target genes (IS*900* and ISMap*02*) were used for real-time PCR diagnosis. Real-time PCR was performed as previously described (53).

### DNA extraction and sequencing.

The feces used for diagnosis were delivered to the laboratory in a refrigerated state and then stored at −80°C. After diagnostic testing, fecal samples selected for sequencing were subjected to DNA extraction. DNA extraction was conducted using a DNeasy PowerSoil kit (Qiagen, Hilden, Germany) according to the manufacturer’s instructions. The extracted DNA was quantified using Quant-IT PicoGreen (Invitrogen). The DNA libraries were constructed according to the Illumina 16S metagenomic sequencing library protocols. Briefly, the V3/V4 region of the bacterial 16S rRNA gene was amplified from 2 ng of input genomic DNA (gDNA) using Herculase II fusion DNA polymerase (Agilent Technologies, Santa Clara, CA) with following primer pair: V3-F, 5′-TCGTCGGCAGCGTCAGATGTGTATAAGAGACAGCCTACGGGNGGCWGCAG-3′, and V4-R, 5′-GTCTCGTGGGCTCGGAGATGTGTATAAGAGACAGGACTACHVGGGTATCTAATCC-3′. Then, additional PCR amplification with the Nextera XT indexed primer was conducted to construct the final library. After every PCR step, purification was conducted using AMPure beads (Agencourt Bioscience, Beverly, MA). The quality of the final product was monitored with a qPCR quantification protocol guide (KAPA library quantification kits for Illumina sequencing platforms) for its quantification and the TapeStation D1000 ScreenTape (Agilent Technologies, Waldbronn, Germany) for its qualification. Finally, the amplicons were sequenced on an Illumina MiSeq 2 × 300-bp paired-end sequencing platform (Macrogen, Daejeon, South Korea).

### Microbial community analysis.

The microbial community was analyzed mainly using the QIIME (Quantitative Insights Into Microbial Ecology) 2 v2021.8 pipeline (54). Raw sequence reads were denoised and ASV tables were generated using DADA2 (55). Taxonomic assignment was conducted using a pretrained naive Bayes classifier on the GreenGenes database, and the relative abundances of bacterial taxa were expressed as percentages of total 16S rRNA sequences. For the microbial diversity (alpha and beta), the feature tables were rarefied to even depths based on the minimum number of features among the samples. The microbial diversity of the samples (alpha diversity) was determined using Pielou’s evenness, observed features, Faith’s phylogenetic diversity, Shannon’s index, and Simpson’s index. Principal-coordinate analysis was performed based on weighted and unweighted UniFrac distances, and the differences in the sample distances between groups were evaluated using PERMANOVA (56). LEfSe analysis was used to identify differentially abundant taxa between the groups with LDA scores of >3.0 and *P* values of <0.05 (57).

Subsequent prediction of the functions of the microbial communities was conducted using PICRUSt2 (Phylogenetic Investigation of Communities by Reconstruction of Unobserved States, version 2.0) (58) and CowPI (23) to predict the functional profile of the microbial communities based on the 16S rRNA gene sequences obtained. Since the web server for CowPI is unavailable, the tool was reconstructed using precalculated files deposited on Zenodo (https://zenodo.org/record/1252858). The predicted metagenomes were obtained from the precalculated KEGG orthologs and classified in a hierarchy using the KEGG pathway metadata. LEfSe analysis was performed using a threshold of an LDA score of >3.0 and a *P* value of <0.05 to identify differentially abundant KEGG pathways.

### Feature selection.

To reduce the dimensionality of the data and select the most significant features in the data set of relative abundance for microbial taxa, five algorithms/tools (ridge regression, LASSO, ElasticNet, Feature Selector [https://github.com/WillKoehrsen/feature-selector], and the filter method) were used. The parameters for each regularization (ridge regression, LASSO, and ElasticNet) were optimized by built-in cross-validation in the Python package Scikit-learn v1.1.1. The hyperparameter values (alpha) for ridge regression, LASSO, and ElasticNet were 0.1, 1.9, and 0.9, respectively. In the case of the filter method, the corr() method of the Python package pandas was used. The threshold value for the correlation was set to 0.5. The parameters for Feature Selector were optimized as follows: missing_threshold, 0.6; correlation_threshold, 0.98; task, classification; eval_metric, auc; and cumulative_importance, 0.95.

### Construction of the machine learning-based classification model.

To build classification models that differentiated MAP-infected individuals from negative individuals cohoused on the positive farm and those on the negative farm, the relative abundance values of the features selected by Feature Selector were used. Four different machine learning algorithms were implemented—LinearSVC (C = 1), KNN (n_neighbors = 3), random forest (n_estimators = 100), and SVM (kernel = linear; C = 1)—using “LinearSVC,” “KNeighborsClassifier,” “RandomForestClassifier,” and “SVC” in the Scikit-learn package. The discriminating performances of the models were measured and compared by values for accuracy and AUC.

### Statistical analysis.

Statistical analyses were conducted using the two-tailed Mann-Whitney U test for 2-group comparisons and the Kruskal-Wallis test for multiple-group comparisons.

### Data availability.

All of the sequence data obtained from the 52 samples in this study were deposited in the sequence read archives of the NCBI under accession number SRP363929.
