# Supplementary material for: A Machine Learning Approach Reveals a Microbiota Signature for Infection with Mycobacterium avium subsp. paratuberculosis in Cattle
Source: Microbiol Spectr. 2023 Jan 19;11(1):e03134-22. doi: 10.1128/spectrum.03134-22 (PMC9927500; doi:10.1128/spectrum.03134-22)
Supplement: Supplemental file 1 — Fig. S1 to S5 and Tables S2 and S3. Download spectrum.03134-22-s0001.pdf, PDF file, 0.3 MB [file spectrum.03134-22-s0001.pdf]

Supplementary figures and tables

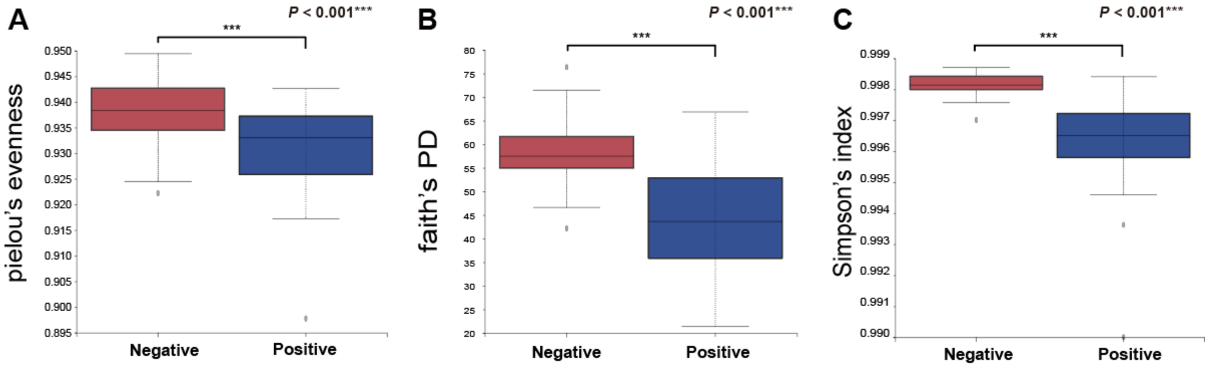

Supplementary Figure S1. Alpha diversity indices for the MAP-negative/-positive group. (A) Evenness index (Pielou's evenness) (B-C) Diversity indices (B: Faith's PD, C: Simpson's index).

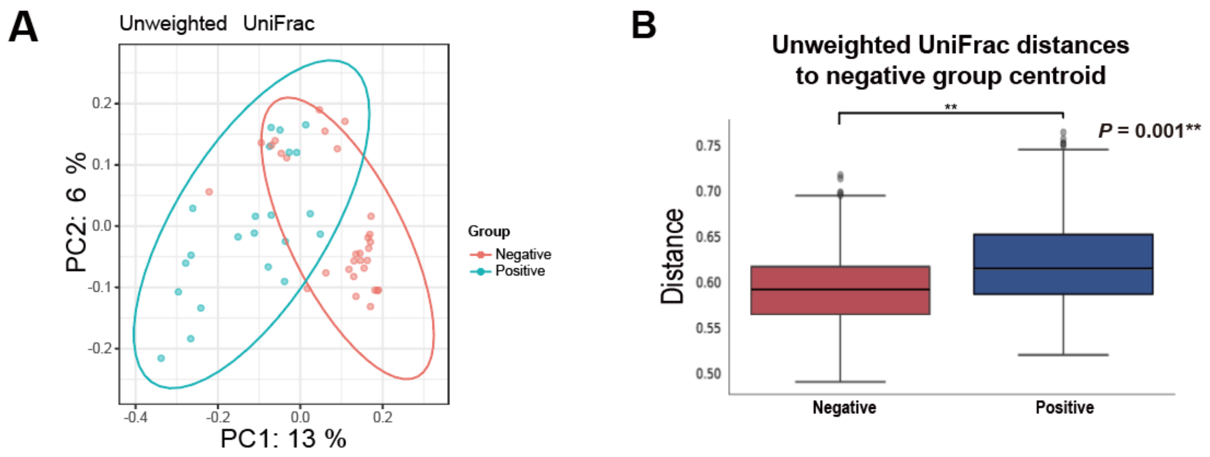

Supplementary Figure S2. Beta diversity based on unweighted UniFrac distances. (A) PCoA plot for unweighted UniFrac distance (B) Box–and-whisker plot for distances from the centroid of the MAP-negative group.

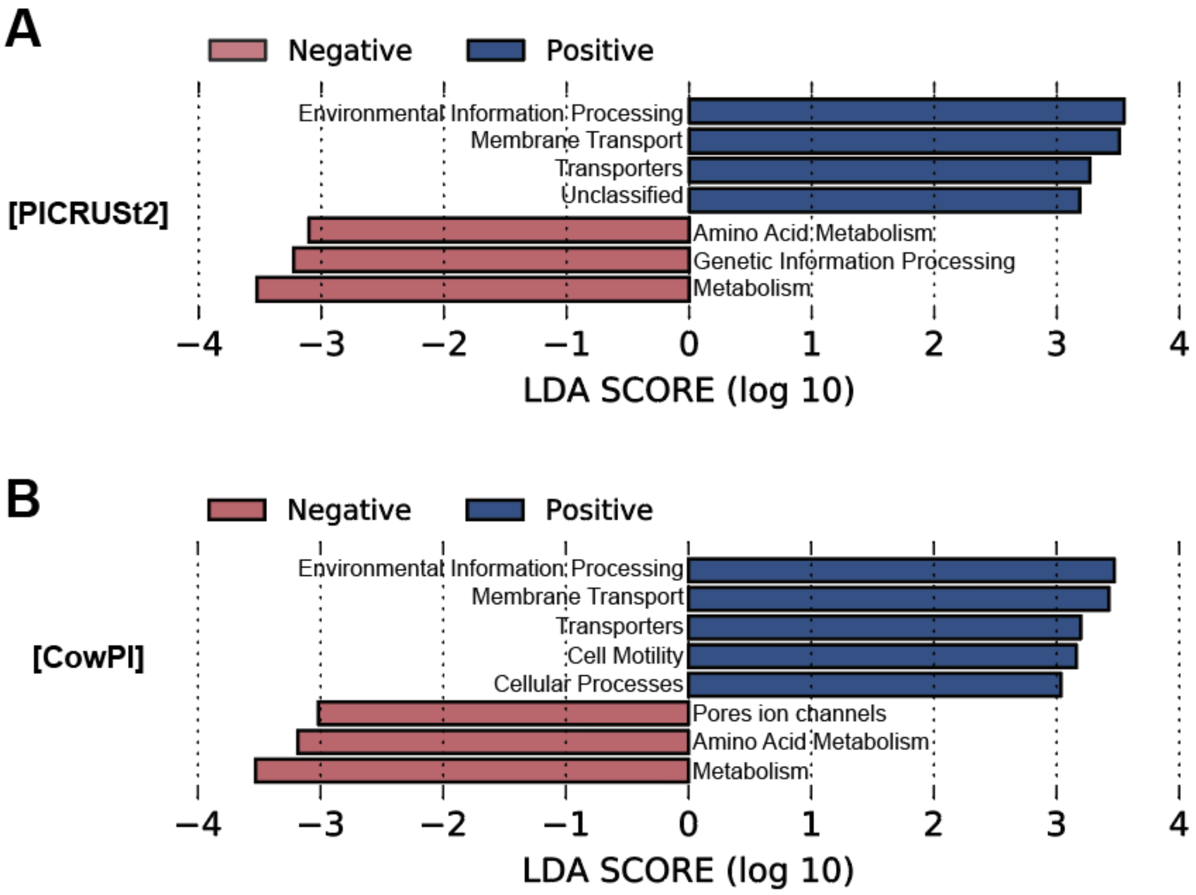

Supplementary Figure S3. Differentially abundant KEGG pathway-mapped metabolic function by MAP infection. (A) The presumptive functions were predicted by PICRUSt2 and (B) CowPI - a rumen microbiome focussed version of PICRUSt. Positive group-enriched pathways are indicated with a positive LDA score (blue), and negative group-enriched pathways with a negative score (red). Only pathways meeting an LDA significance threshold of >3 are shown.

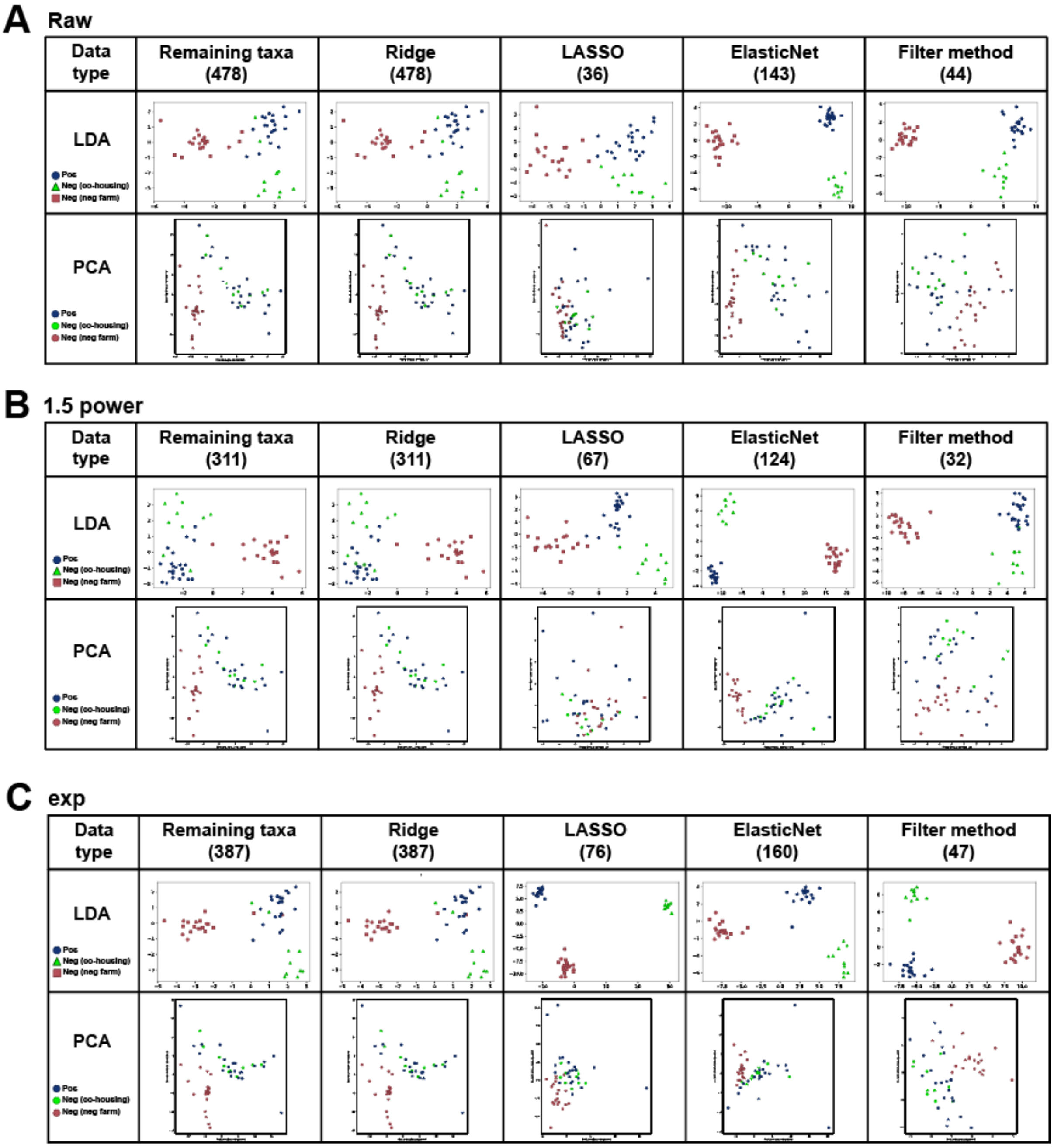

Supplementary Figure S4. Selection of microbial features using four feature selection algorithms/tools with three different types of transformed values. (A) LDA and PCA plots after selecting microbial features from the quasi/constant value-removed remaining taxa dataset, (B) its 1.5 power-transformed dataset, and (C) exponential-transformed dataset. All numeric values in parentheses indicate the number of features selected by each algorithm/tool. The Ridge method was excluded for visualization since there was little reduction after selecting the features.

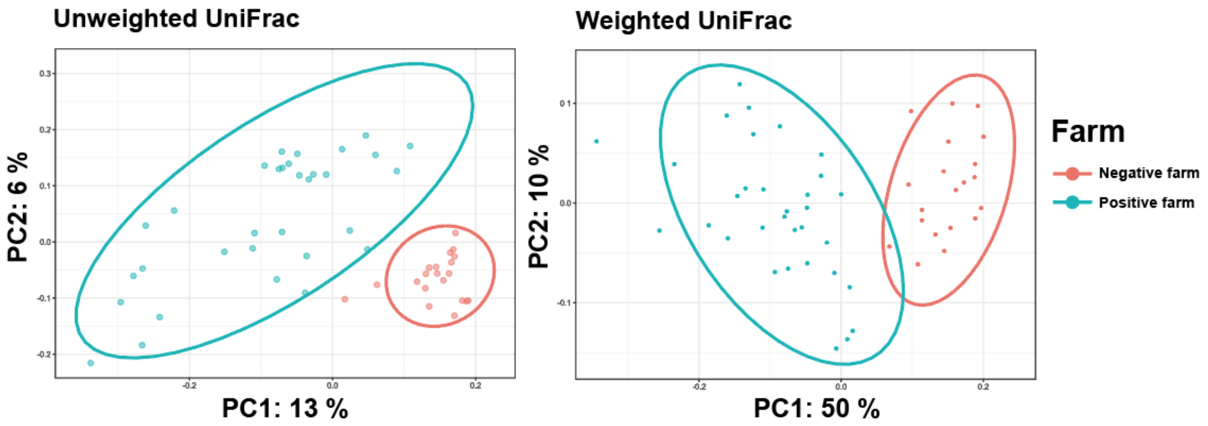

Supplementary Figure S5. PCoA plot for beta diversity based on un-/weighted UniFrac distances according to farm. Red circles and cyan circles indicate animals in the negative farm and the positive farm, respectively.

Supplementary Table S1. General information including diagnostic results and taxa composition of all 52 samples

[attached excel file]

Supplementary Table S2. The Calinski–Harabasz index and the Silhouette score for LDA clustering of the selected features using five feature selection methods

	**Calinski-Harabasz index**			**Silhouette score**		
Data type	Raw	1.5 power	exp	Raw	1.5 power	exp
Original data	128.824			0.611		
Remaining taxa	129.558	211.142	196.303	0.640	0.653	0.706
Ridge	129.558	211.142	196.303	0.640	0.653	0.706
LASSO	76.815	128.144	3467.708	0.512	0.630	0.908
ElasticNet	1122.101	2553.817	581.362	0.852	0.867	0.824
Feature Selector	91.371	78.317	124.327	0.457	0.503	0.528
Filter method	877.581	609.471	798.224	0.770	0.725	0.834

Supplementary Table S3. Accuracy and AUC values of random forest models based on a combination of selected microbial features (M) and conventional diagnostic tools.

**Type**	**M**		**M + PCR**		**M + ELISA**		**M + both**		***P* value**
	Acc	AUC	Acc	AUC	Acc	AUC	Acc	AUC	
**Raw**	0.89±0.	0.93±0.	0.85±0.	0.92±0.	0.83±0.	0.93±0.	0.84±0.	0.92±0.	0.98

	08	05	12	08	08	05	07	08	
**1.5 power**	0.84±0.07	0.96±0.04	0.89±0.06	0.96±0.05	0.85±0.06	0.96±0.04	0.87±0.10	0.94±0.07	0.63
**exp**	0.86±0.07	0.94±0.05	0.86±0.06	0.95±0.06	0.84±0.09	0.96±0.04	0.89±0.11	0.96±0.07	0.27

All values are the mean±SD of values for model accuracy (Acc) and AUC of random forest models with 10-fold cross-validation (training set: n=49, testing set: n=5) based on the labeled information of each sample. The models were constructed using selected microbial features (M) or their combination with the results of other conventional diagnostic tools (e.g. PCR, ELISA, or both) for every data type (Raw, 1.5 power, and exp). The column “*P* value” indicates the *p* values of Kruskal–Wallis test for each row.
